# Prospective Analysis of Food Consumption and Nutritional Status and the Impact on the Dietary Inflammatory Index in Women with Breast Cancer during Chemotherapy

**DOI:** 10.3390/nu11112610

**Published:** 2019-11-01

**Authors:** Isis Danyelle Dias Custódio, Fernanda de Paula Franco, Eduarda da Costa Marinho, Taísa Sabrina Silva Pereira, Mariana Tavares Miranda Lima, Maria del Carmen Bisi Molina, Nitin Shivappa, James R. Hebert, Carlos Eduardo Paiva, Yara Cristina de Paiva Maia

**Affiliations:** 1 Graduate Program in Health Sciences, Federal University of Uberlandia, Uberlandia, 38405-320 Minas Gerais, Brazil; isisdanyelle@yahoo.com.br (I.D.D.C.); eduardacosta.marinho@gmail.com (E.d.C.M.); tmmariana@hotmail.com (M.T.M.L.); 2 Nutrition Course, Medical Faculty, Federal University of Uberlandia, Uberlandia, 38405-320 Minas Gerais, Brazil; fer_nandinhafranco@hotmail.com; 3 Nutrition, Department of Health Sciences, University of the Americas Puebla, Cholula, 72810 Puebla, Mexico; taisa.sabrina@hotmail.com; 4 Graduate Program in Nutrition and Health, Federal University of Espirito Santo, Vitoria, 29047-105 Espirito Santo, Brazil; mdcarmen2007@gmail.com; 5 Nutrition Course, Federal University of Espirito Santo, Vitoria, 29047-105 Espirito Santo, Brazil; 6 South Carolina Statewide Cancer Prevention and Control Program, University of South Carolina, Columbia, SC 29208, USA; shivappa@email.sc.edu (N.S.); JHEBERT@mailbox.sc.edu (J.R.H.); 7 Department of Epidemiology and Biostatistics, University of South Carolina, Columbia, SC 29208, USA; 8 Connecting Health Innovations LLC, Columbia, SC 29201, USA; 9 Department of Clinical Oncology, Graduate Program in Oncology, Barretos, 14784-400 Sao Paulo, Brazil; drcarlosnap@gmail.com; 10 Palliative Care and Quality of Life Research Group (GPQual), Pio XII Foundation—Barretos Cancer Hospital, Barretos, 14784-400 Sao Paulo, Brazil

**Keywords:** food consumption, inflammation, nutritional status, breast neoplasms, diet, food and nutrition, antineoplastic agents, cancer, dietary inflammatory index

## Abstract

Considering the implications of adverse effects of chemotherapy (CT) and the potential impact of diet on patients’ recovery, this study aimed to prospectively evaluate the association between the consumption of food groups, patients’ Dietary Inflammatory Index (DII^®^) scores, and their nutritional status. Anthropometric and dietary assessments of 55 women with breast cancer (BC) were performed at three time points. T0 is the time point after the first CT cycle, T1 is the time point after the intermediate CT cycle, and T2 is the time point after the last CT cycle. We identified a significant increase in weight, body mass index, and waist circumference during CT. Consumption of poultry and eggs was higher in T1 when compared to T2, while consumption of total fruit and total vegetables was higher at T0 compared to T1 and T2. The diet became more pro-inflammatory over the course of treatment (X^2^_(2)_ = 61.127), and was related to higher abdominal adiposity. Total fruit (T0: R^2^ = 0.208, T1: R^2^ = 0.095, T2: R^2^ = 0.120) and total vegetable consumption (T0: R^2^ = 0.284, T1: R^2^ = 0.365, T2: R^2^ = 0.580) predicted DII^®^ change at the three-time points. Meanwhile, consumption of total grains was significantly associated only with T1 (R^2^ = 0.084) and T2 (R^2^ = 0.118), and consumption of simple sugars was significantly associated only with T0 (R^2^ = 0.137) and T1 (R^2^ = 0.126). Changes in food consumption led to an increase in the inflammatory profile of the diet, suggesting the necessity to improve the guidelines during and after CT. These results reinforce the need to promote healthier eating practices in concert with maintaining a healthy nutritional status in women with BC treated with CT.

## 1. Introduction

Breast cancer (BC) is the major cause of cancer death in women and its incidence has increased rapidly [1]. The world estimate for 2035 is for 2.6 million cases and 846 thousand deaths from this disease [2]. Among treatment options, chemotherapy (CT) makes it possible to cure some tumors and to treat non-detectable metastases in advance of clinical presentation [3]. However, the use of drugs with a systemic cytotoxic action affects both tumor cells and normal cells, which can cause toxicity and adverse effects, impacting food consumption and nutritional status [4].

During CT sessions, patients may present with various adverse side effects, such as nausea, pain, fatigue, and changes in perception of flavor and smell, which may decrease food palatability and consequently, result in inadequate food intake [5]. According to recent data from our research group, in women with BC, CT interferes negatively with diet and perceptions of the organoleptic properties of food, as well as nutritional status and quality of life [6,7,8,9]. Despite the adverse effects of CT on dietary adequacy, weight gain is very common in women with BC; this may be influenced by relatively common treatment with steroid drugs, menopausal status, and tumor characteristics [5,10]. It is important to emphasize that a patient’s initial weight or excess weight gain after cancer diagnosis negatively impacts prognosis, quality of life, and survival of women with BC [11,12,13]. This appears to be due, in part, to changes in levels of chronic, systemic inflammation, as indicated by inflammatory markers [14,15].

In addition to being overweight, another factor capable of modulating inflammation is diet [16,17]. The Western dietary pattern, which is rich in red meat, full-fat dairy products, and refined grains, is related to the increase of inflammatory markers such as C-reactive protein (CRP) and interleukin-6 (IL-6) [17]. Conversely, the Mediterranean diet, which is characterized by high consumption of fruit, vegetables, whole grains, and fish; low consumption of red meat and butter; and moderate consumption of alcohol and olive oil, is related to the decrease of these inflammatory markers [17]. Inflammation may also increase the risk of several diseases such as diabetes, cardiovascular, osteoarticular, and intestinal diseases and even cancer (e.g., second primaries) [18].

Cavicchia et al. [19] developed and validated a dietary index capable of predicting the level of inflammation and the results of cancer and other diseases and health events. The second generation of the Dietary Inflammatory Index (DII^®^), developed by Shivappa et al. [20], identified a total of 45 dietary parameters associated with six inflammatory biomarkers.

CT is a treatment that negatively interferes with nutritional status and food consumption, both in quantitative and qualitative terms [21,22], and such changes may, in turn, interfere with the inflammatory profile. Therefore, it is relevant to investigate the relationship between food consumption, nutritional status, and inflammation throughout treatment. We hypothesize that throughout CT, the diet of women with BC becomes more pro-inflammatory as a result of the increase in the consumption of calorie-dense foods that are associated with higher DII^®^ scores (more pro-inflammatory diet). Thus, this study aimed to evaluate, in a sample of women with BC receiving CT, the association between the average consumption of food groups (grams/day) and the DII^®^ score, as well as changes in nutritional status throughout CT treatment.

## 2. Materials and Methods

### 2.1. Study Design and Ethical Aspects

The study was carried out from August 2014 to October 2015 in women with BC receiving CT treatments at Clinics Hospital of the Federal University of Uberlandia, Minas Gerais, Brazil. Participants agreed to sequential evaluations, and follow-up time ranged from 4 to 6 months, according to the chemotherapeutic regimen. Assessments were performed at three time points: T0 is the time point after the first CT cycle, T1 is the time point after the intermediate CT cycle, and T2 is the time point after the last CT cycle.

As different CT regimens display distinct numbers of cycles, we choose, as T1, the third CT cycle when FAC (5-fluorouracil, adriamycin, and cyclophosphamide on day 1, repeated every 21 days for a total of 6 cycles) and CMF (cyclophosphamide, methotrexate, and 5-fluorouracil on day 1, repeated every 21 days, for a total of 6 cycles) were used, and the fourth CT cycle when an AC-docetaxel (4 cycles of adriamycin and cyclophosphamide followed by 4 cycles of docetaxel, both repeated every 21 days) and AC-paclitaxel (4 cycles of adriamycin and cyclophosphamide every 21 days followed by 12 sequential weeks of paclitaxel) regimen was used.

This prospective study was approved by the Human Research Ethics Committee (no. 721,977/14, addendum no. 1,111,998/15), and complies with ethical principles and norms of the Declaration of Helsinki and Resolution CNS 466/12.

### 2.2. Sample Size and Elegibility Criteria

We used the G*Power software, version 3.1 (Düsseldorf, Germany) to calculate the sample size [23]. An F test was conducted using ANOVA repeated measures. Based on an effect size f of 0.25, an alpha level of 0.05, 95% power, one group of individuals, three measurements, and a 20% adjustment for possible losses of degrees of freedom, we determined that a minimum of 52 women were needed at T0.

The inclusion criteria were females aged 18 years or older that had been diagnosed with primary BC, who were in their first cycle of CT, and had both the verbal and cognitive ability to participate. 

Women with any of the following characteristics were excluded: under 18 years of age, had a change in treatment due to toxicity or disease recurrence, had a primary tumor site other than the breast, had undergone an anticancer treatment that did not include CT, were not in the first cycle of CT, and had either verbal or cognitive impediments.

All eligible women who had been seen during the study period were invited to participate in the study even if they were waiting for medical consultation in the oncology department. Once informed about the project, volunteers who were eligible to participate agreed to enroll by signing the voluntary consent form.

Data on socioeconomic, clinical, and therapeutic status were collected during interviews and from medical records.

### 2.3. Anthropometric Assessment

Measurements were taken by an experienced nutritionist using a protocol of the World Health Organization (WHO) [24]. Volunteers were requested to wear only light clothes and be barefoot. Their body weight (kg) and height (m) were measured to calculate the body mass index (BMI in kg/m^2^). A calibrated mechanical scale (model P-150C; Líder Balanças, Sao Paulo, Brazil) was used to measure weight, which had a sensitivity of 100 g; for height, a vertical stadiometer (model P-150C; Líder Balanças) with a 1-mm precision scale was used. The BMI was classified as recommended by the WHO [25] for the adult population (age >20 years and <60 years), with underweight: <18.5 kg/m^2^, normal weight: 18.5–24.9 kg/m^2^, overweight: 25.0–29.9 kg/m^2^, grade I obesity: 30.0–34.9 kg/m^2^, grade II obesity: 35.0–39.9 kg/m^2^, and grade III obesity ≥40.0 kg/m^2^; and by Lipschitz [26] for the elderly (≥60 years), with underweight: <21.9 kg/m^2^, normal weight: 22–27 kg/m^2^, overweight: >27 kg/m^2^. A flexible and inelastic tape with a 1-mm precision was used to measure waist circumference (WC) and hip circumference (HC), using a protocol from Lohman, Roche, and Martorell [27]. These measures were used to compute the waist-to-hip ratio (WHR) and the waist-to-height ratio (WHtR). In order to classify the risk of metabolic complications associated with obesity, we used these cut-off points proposed by the WHO [25]: >80 cm for WC; and >0.85 for WHR. To assess WHtR, the cut-off point of ≥0.5 established by Ashwell and Hsieh [28] was adopted, which indicates an excess of abdominal adiposity.

### 2.4. Dietary Assessment

Dietary assessment was performed using data obtained from the telephone application of three 24-hour dietary recalls (24HRs) on two non-consecutive weekdays and one on the weekend at each CT time; totaling nine 24HRs per participant. Telephone calls were made starting from the day after the infusion of CT and before the next appointment (Δt = 21 days), preferably in the second week after the infusion to avoid the acute effect of CT. From these 24HRs, we calculated the consumption of the food groups in grams per day (g/day) and, ultimately, the DII^®^ score.

Dietpro^®^ software version 5.7 (AS Sistemas, Viçosa, MG, Brazil) was used to convert measurements from the 24HRs in grams or milliliters and these data were entered into a Microsoft Excel version 1908 (Redmond, WA, USA) spreadsheet. Consumption data, in g/day, included the following food groups: total fruit (including fruit and natural fruit juices); total vegetables (raw, cooked, salted); total grains (refined cereals, roots, tubers, bakery products, except whole grains); whole grains; fish; red meat (bovine and porcine); poultry and eggs; milk and dairy products; beans; vegetable oils; and simple sugars (including the additional sugar and the sugar of soft drinks, sweets, chocolate, and honey). The quantification of nutrients was performed using Nutrition Data System for Research (NDSR) software version 2010 (Minneapolis, MN, USA), using the table from the United States Department of Agriculture [29] as a reference, and for foods not found in this table, the Brazilian Food Composition Table [30] was used in addition to the software output.

We calculated the DII^®^ score using the method established by Shivappa et al. [20]. In this study, a total of 28 food parameters were used for the calculation of DII^®^: vitamin B12, vitamin B6, beta-carotene, carbohydrate, cholesterol, energy, fat, fiber, iron, magnesium, monounsaturated fatty acids (MUFAs) and polyunsaturated fatty acids (PUFAs), niacin, omega 3, omega 6, protein, riboflavin, saturated fat, selenium, thiamine, vitamin C, vitamin D, vitamin E, zinc, isoflavone, trans fat, vitamin A, and folic acid. 

In order to reduce the intra- and inter-individual variability of food consumption, before the calculation of the DII^®^, the energy and nutrient consumption were deattenuated [31]. Subsequently, these variables were adjusted by the mean energy of the sample using the residual method [32]. 

### 2.5. Statistical Analysis

Data were analyzed using SPSS^®^ software, version 21.0 (SPSS Inc., Chicago, IL, USA). Initially, normality was evaluated using the Kolmogorov–Smirnov test. According to variable distributions, parametric or non-parametric tests were performed, and the results were expressed as mean ± standard deviation, or as a median with 25th and 75th percentiles.

The one-way ANOVA test with repeated measures and Bonferroni post-hoc, or the non-parametric Friedman with multiple comparison tests, were used throughout the treatment (T0, T1, and T2) for the analysis of the DII^®^ score, anthropometric measures, and mean consumption of food groups (g/day). An independent-samples t-test and Mann–Whitney test were used to compare means and mean ranks, respectively, of anthropometric measures categorized as below or equal/above the median DII^®^ score. Simple linear regression analyses were performed to verify the predictive capacity of the mean consumption of each food group (independent variable) in relation to the DII^®^ score (dependent variable). Extreme values/outliers were removed for this statistical test. Also, correlations were made between the anthropometric variables and DII^®^ scores at the three CT time points using a Pearson or Spearman correlation. The DII^®^ was evaluated as a continuous variable and also as a categorical variable (0 = below the median and 1 = equal or above median). The 95% confidence interval (CI) and *p*-values were also calculated.

## 3. Results

Fifty-five women with BC receiving CT participated in the present study. The population has already been detailed in a previous study [9]. Clinical, therapeutic, and socio-demographic characteristics are shown in Table 1. Most (96.4%, *n* = 53) of the patients had invasive ductal carcinoma and 61.8% (*n* = 34) were post-menopausal. A total of 32 (58.2%) patients underwent adjuvant CT and the majority (60%, *n* = 33) received the AC➔docetaxel regimen. 

It can be seen in Table 2 that there was no statistical difference between the anthropometric measures categorized as below or above median DII^®^ score. However, weight, BMI, and WC differed statistically across CT times. It should be noted that these results were published by Custódio et al. [7]. However, they are presented here once again so that it is possible to identify the change between the CT times compared with the correlations between these variables and the DII^®^ score. Weight, BMI, and WC increased significantly (*p* = 0.008, *p* = 0.009, and *p* = 0.03, respectively), indicating a worsening of nutritional status when compared to the beginning (T0) and ending of CT (T2), although this increase in WC had not been detected in multiple comparisons test (Table 2).

Correlations between DII^®^ and the WHtR and WHR variables were weak and positive during (T1) and after CT (T2), indicating that those women displaying a greater accumulation of adipose tissue in the abdominal area reported a more pro-inflammatory diet at the time points mentioned.

**Figure 1:**
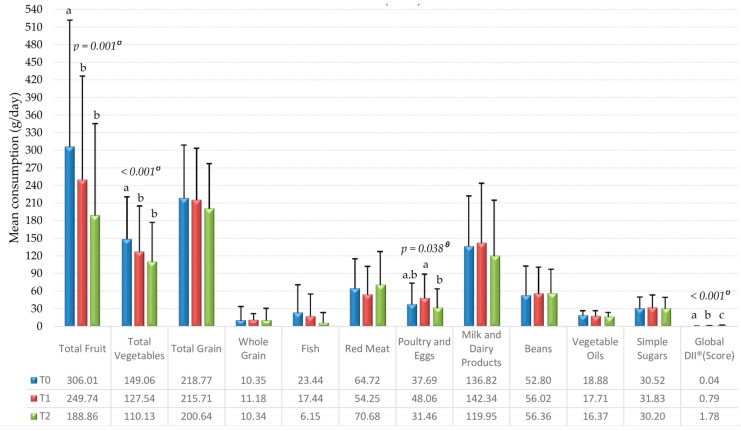
Consumption of food groups (g/day) and dietary inflammatory index (DII^®^) of women with breast cancer in chemotherapy (CT) attended at Clinics Hospital at Federal University of Uberlandia, Minas Gerais, Brazil, 2014–2015 (*n* = 55). T0, period after the administration of the first cycle of CT; T1, period after administration of the intermediate cycle; T2, period after administration of the last CT cycle; ᶿ One-way ANOVA with repeated measures + Bonferroni post-hoc; ᶷ Friedman + Multiple comparisons test; Means horizontally followed by different letters differ statistically as post-hoc test at 5% probability.

In relation to food consumption, Figure 1 shows the mean consumption of food groups over the three CT time points. We identified the effect of CT time on the DII^®^ score, which differed significantly (X^2^_(2)_ = 61.127; *p* < 0.001) between the three time points, indicating that the diet became more pro-inflammatory throughout the treatment (median (p25–p75): T0, 0.14 (−0.77–0.74); T1, 0.86 (−0.06–1.64); T2, 1.89 (1.17–2.42)). CT time also impacted the consumption of poultry and eggs (F(2,108) = 3.358, *p* = 0.04), with consumption differing between T1 and T2, i.e., during CT, the consumption was significantly higher than at the end of CT. The consumption of the total fruit (X^2^_(2)_ = 13.237, *p* = 0.001) and total vegetables (X^2^_(2)_ = 17.491, *p* < 0.001) food groups also showed a difference between CT times, and at the beginning of the treatment, the consumption of these two groups (total fruit and total vegetables) was higher than the consumption at T1 and T2 (Figure 1).

Results of linear regression using DII^®^ as a dependent variable indicated that total fruit (T0: F(1,50) = 13.127, *p* = 0.001, R^2^ = 0.208; T1: F(1,52) = 5.488, *p* < 0.001, R^2^ = 0.095; T2: F(1,50) = 6.841, *p* = 0.012, R^2^ = 0.120) and total vegetables (T0: F(1,50) = 19.851, *p* < 0.001, R^2^ = 0.284; T1: F(1,49) = 28.176, *p* < 0.001, R^2^ = 0.365; T2: F(1,50) = 68.940, *p* < 0.001, R^2^ = 0.580) were able to predict a DII^®^ change at the three time points of CT. Meanwhile, the total grains group was only significant at T1 and T2 (T1: F(1,52) = 4.783, *p* = 0.033, R^2^ = 0.084; T2: F(1,50) = 6.677, *p* = 0.013, R^2^ = 0.118), and simple sugars was only significant at T0 and T1 (T0: F(1,51) = 8.124, *p* = 0.006, R^2^ = 0.137; T1: F(1,52) = 7.523, *p* = 0.008, R^2^ = 0.126) (Table 3).

## 4. Discussion

The current study investigated the relationship between the inflammatory profile of the diet and the consumption of food groups in women with BC undergoing CT, as well as the relationship between the inflammatory profile of the diet and women’s overall nutritional status. Significant changes in the nutritional status and food consumption of women with BC during CT were observed. Weight, BMI, and WC differed significantly between CT times, indicating a worsening of the nutritional status, with the WHtR and WHR variables presenting weak, positive correlations in relation to DII^®^, during (T1) and after CT (T2). This indicates that the greater the accumulation of adipose tissue in the abdominal area, the more pro-inflammatory the diet. The consumption of poultry and eggs was significantly higher during CT (T1) when compared to the end of the treatment (T2), while total fruit and total vegetables were consumed at higher rates at the beginning (T0) in relation to T1 and T2. Significant change was also observed in relation to the DII^®^ score, which increased considerably, indicating that the diet of the women became more pro-inflammatory in the course of the treatment. The DII^®^ score predicted the consumption of total fruit and total vegetables at all three time points; while the DII^®^ predicted total grain consumption in T1 and T2; and simple sugars in T0 and T1.

During CT, there is a reduction in the olfactory and gustatory functions, negatively impacting the sensations of both satisfaction and comfort in relation to food [21,33]; although such functions are restored three months after the sessions [34]. Besides these factors reducing the pleasure of meals, CT sessions can result in other important adverse effects, such as nausea and vomiting, which also would have a clear impact on food consumption. In the study by Steinbach et al. [34], salty flavor was most affected by changes in taste sensitivity, while Marinho et al. [8] found that the change in sensory perception favored a preference for sugars at the three time points of CT.

In the same sample of women with BC from the present study, Custódio et al. [7] identified a significant reduction in the Brazilian Healthy Eating Index Revised (BHEI-R) score for the total fruit group and the dark green vegetables and orange vegetables and legumes group. The results presented here corroborate these findings; the mean consumption of total fruit and total vegetables was in fact significantly affected throughout the treatment. The change in palatability may be associated with the reduction of consumption of these food groups, considering that they are perceived as less palatable due to their reduced sugar and fat content.

The consumption of poultry and eggs was inversely related relative to red meat, signaling a variation of the protein option through T0 and T1. Marinho et al. [8] evaluated the appetite score for meat, poultry, fish, and eggs during CT as a single group, and it is not possible to state whether this consumption behavior is related to appetite or to a reduction in the preference and increase in the aversion to meat in general throughout the treatment, or due to the availability and affordability. However, although the reasons have not been established, it is especially relevant to understand the consumption of food that are sources of nutrients such as iron, especially heme iron, given its greater bioavailability when faced with the anemia frequently diagnosed among patients undergoing antineoplastic treatment [35].

The consumption of total grains and simple sugars did not change significantly over the CT period. However, the consumption of these food groups was still sufficiently higher to predict an increase in the DII^®^ score. The diet of these women became more pro-inflammatory because of the high consumption of these predominantly pro-inflammatory food groups, as well as the low consumption of fruit and vegetables, which are rich in anti-inflammatory nutrients, thus illustrating how dietary choices can impact the dietary profile.

The diet plays an important role in the modulation of inflammatory responses [16]. The adoption of a Mediterranean dietary pattern contributes to the reduction of inflammation [36], and in turn, likely associated diseases, such as vascular diseases [36,37] and cancer [38]. In contrast, a more pro-inflammatory diet may favor cardiovascular diseases, which are considered to be the main cause of death among women surviving BC [39].

Pierce et al. [40] present evidence that chronic inflammation is associated with reduced overall survival and reduced disease-free survival in women with BC. Pro-inflammatory markers are correlated with tumor growth and metastasis and may be used as prognostic indicators [41].

CT causes body weight gain, with excess adipose tissue being a concern both in the short and medium term [42]. This is important in light of the fact that obesity is associated with an increased risk of important diseases, such as hypertension, atherosclerosis, and myocardial infarction [43], and eight types of cancer, including the post-menopausal BC [1]. Obesity leads to adipokine imbalance, promoting a chronic, low-grade inflammatory state, and consequently, likely systemic metabolic dysfunction [43]. BC survivors have a higher mortality risk for chronic diseases other than cancer, emphasizing the importance of diet quality and the maintenance of a healthy nutritional status for survival, regardless of the stage of the disease [44].

We showed that women with BC began treatment overweight, and the WHR and WHtR ratios were positively correlated with DII^®^ scores. A study carried out with adolescent women with different adiposity levels demonstrated that an increase in WC and WHtR was proportional to an increase in both BMI and body adipose tissue. In addition, a relationship was found between body mass and total adiposity and with central and peripheral adipose tissue [45]. The study found positive correlations between measures of central adiposity and the risk of BC in post-menopausal women [46]. A recent meta-analysis showed a linear association between BMI and BC risk, and reported that this risk increased 2% for each increase of 5 kg/m^2^ in BMI among post-menopausal women [47].

It is important to consider the limitations of this study. These include the non-use of electrical bioimpedance in the evaluation of the participants’ body composition. Increased body weight, BMI, and WC were identified; however, it was not possible to state that such results were due to a gain in adipose tissue or water retention, an important consideration given that the CT included the use of corticosteroids. Weight gain is frequent among women receiving CT [42,48,49], and this is typically associated with an increase in the percentage of body adiposity [1,48]. Another explanation is that there may be a reduction in physical activity among women with BC after diagnosis, accelerating this expected weight gain [50].

Although the 24HRs is considered state-of-the-art for dietary assessment, it is not without error [51]. Clearly, the ability to report accurately depends on a number of factors including the ability of an individual to recall their food consumption without either underestimation due to omissions or overestimation due to intrusions. In an attempt to minimize such bias, interviews were carried out by properly trained nutritionists. Nine 24HRs were conducted for each participant (three at each of the time points evaluated) and nutrient intakes were deattenuated and adjusted for total energy intake to reduce the intrinsic variability of consumption.

## 5. Conclusions

The results show that women with BC receiving CT presented significant changes in nutritional status and the consumption of total fruit, total vegetables, poultry, and eggs. In addition, a significant change was observed in relation to DII^®^, with a significant increase during the treatment, which was related to the intake of the total fruit, total vegetables, total grains, and simple sugars groups, as well as higher abdominal adiposity.

Considering that the increase in the inflammatory profile of the diet was caused by such changes in food consumption, it may be necessary to stress the dietary guidelines, both during and after treatment, such that BC survivors may adopt healthier eating practices. The adoption of a diet with adequate nutritional quality, associated with maintaining healthy weight, demonstrates the risk reduction of relapse and comorbidities. To put the current study’s results into context, it is important to note that previous work with macrobiotics (a Japanese-inspired low-DII^®^ diet) [52,53] indicates a possible reduction in the risk of recurrence [54].

Based on the results presented, it is reasonable to encourage female BC patients on CT to adopt a low-DII^®^ diet to improve their prognosis and hasten recovery from the disease. 

## Figures and Tables

**Table 1:** Clinical, therapeutic, and socio-demographic characteristics of women with breast cancer in chemotherapy seen at Clinics Hospital of the Federal University of Uberlandia, Minas Gerais, Brazil, 2014–2015 (*n* = 55).

Variable	*n* or Mean ± SD	%
Age	51.5 ± 10.1	100.0
**Marital status**		
With partner	33	60.0
Without partner	22	40.0
**Income (R$^1^)**		
≤2 minimum wage	29	52.7
>2 minimum wage	26	47.3
**Education**		
≤9 years of study	24	43.6
9 to 12 years of study	18	32.7
>12 years of study	12	21.8
**Tumoral Subtype**		
Invasive ductal carcinoma	53	96.4
Invasive lobular carcinoma	2	3.6
**Menopausal Status**		
Pre-menopausal	21	38.2
Post-menopausal	34	61.8
**Surgery**		
Conservative	24	43.6
Mastectomy	8	14.6
Did not undergo surgery (neoadjuvant)	23	41.8
**Chemotherapy**		
Adjuvant	32	58.2
Neoadjuvant	23	41.8
**Chemotherapy Regimen**		
AC → Docetaxel	33	60.0
AC → Paclitaxel	8	14.6
FAC	9	16.4
CMF	5	9.1

SD—standard deviation, ^1^ Monthly minimum wage, R$880.00; NR, not reported; G1—well-differentiated tumor (low grade); G2—moderately differentiated tumor (intermediate grade); G3—poorly differentiated tumor (high grade); AC→Docetaxel—adriamycin and cyclophosphamide followed by docetaxel; AC→Paclitaxel—adriamycin and cyclophosphamide followed by paclitaxel; FAC—5-fluorouracil, adriamycin, and cyclophosphamide; CMF—cyclophosphamide, methotrexate, and 5-fluorouracil.

**Table 2:** Nutritional status and Dietary Inflammatory Index (DII^®^) of women with breast cancer in chemotherapy seen at Clinics Hospital of the Federal University of Uberlandia, Minas Gerais, Brazil, 2014–2015 (n = 55).

Variables	Median DII^®^ T0 (0.141)		*p*	Median DII^®^ T1 (0.864)		*p*	Median DII^®^ T2 (1.888)		*p*	T0	T1	T2	*p*	DII^®^		
	Below	Equal/Above		Below	Equal/Above		Below	Equal/Above						T0	T1	T2
Weight (kg)	62.5 (56.1–80.6)	73.2 (60.1–85.3)	0.08	65.2 (58.7–86.3)	67.3 (58.2–83.9)	0.97	64.6 (58.2–85.1)	68.0 (59.8–81.5)	0.66	66.1 (58.5–84.1)	67.2 (58.6–83.9) ^a.b^	66.4 (58.5–83.1) ^b^	*0.008 ᶷ*	0.205 ^†^	0.068 ^†^	0.143 ^†^
BMI (kg/m^2^)	27.0 ± 6.7	29.7 ± 5.9	0.12	25.8 (23.7–32.4)	27.6 (23.7–35.0)	0.71	25.9 (23.7–33.6)	26.6 (24.1–34.2)	0.64	26.4 (23.5–33.7) ^a^	26.3 (23.8–33.6) ^a.b^	26.5 (23.9–33.3) ^b^	*0.009 ᶷ*	0.190 ^†^	0.159 ^†^	0.209 ^†^
WC (cm)	87.1 ± 16.4	94.2 ± 14.4	0.09	90.2 ± 16.5	92.2 ± 14.8	0.63	90.1 ± 16.3	92.0 ± 14.4	0.64	86.5 (78.5–105.0) ^a^	88.0 (79.0–103.0) ^a^	87.0 (80.0–103.5) ^a^	*0.030 ᶷ*	0.165 ^†^	0.217 ^‡^	0.218 ^†^
WHR	0.84 ± 0.1	0.87 ± 0.1	0.10	0.84 ± 0.07	0.86 ± 0.07	0.40	0.84 ± 0.07	0.86 ± 0.07	0.35	0.9 ± 0.1	0.9 ± 0.1	0.9 ± 0.1	0.221 ᶿ	0.165 ^†^	0.252 ^‡^	0.298 ^‡^ *
WHtR	0.56 ± 0.1	0.59 ± 0.1	0.16	0.57 ± 0.1	0.59 ± 0.1	0.43	0.57 ± 0.1	0.59 ± 0.1	0.49	0.6 (0.5–0.7)	0.6 (0.5–0.7)	0.6 (0.5–0.7)	0.761 ᶷ	0.153 ^†^	0.263 ^‡ **^	0.247 ^†^

SD—standard deviation, T0—time point after the first CT cycle, T1—time point after the intermediate CT cycle, T2—time point after the last CT cycle, BMI—body mass index, WC—waist circumference, WHR—waist-to-hip ratio, WHtR—waist-to-height ratio. Independent-samples t-test and Mann–Whitney test were used to compare means and mean ranks, respectively, of anthropometric measures categorized as below or equal/above median DII^®^ score, according to the normality. ᶿ One-way ANOVA with repeated measures plus Bonferroni post-hoc, ᶷ Friedman plus multiple comparisons test, ^†^ Spearman correlation, ^‡^ Pearson correlation. Means/medians horizontally followed by different letters differ statistically in the post-hoc test at 5% probability. * *p* = 0.027, ** *p* = 0.026.

**Table 3:** Association between Dietary Inflammatory Index (DII^®^) and the consumption of food groups (g/day) of women with breast cancer in chemotherapy (CT) seen at Clinics Hospital at Federal University of Uberlandia, Minas Gerais, Brazil, 2014–2015.

Food Groups (g/Day)	T0				T1				T2			
	*n*	B	SE	*p*	*n*	B	SE	*p*	*n*	B	SE	*p*
Total Fruit	52	−0.003	0.001	0.001	54	−0.002	0.001	0.02	52	−0.002	0.001	0.01
Total Vegetables	52	−0.009	0.002	<0.001	51	−0.011	0.002	<0.001	52	−0.010	0.001	<0.001
Total Grains	52	0.004	0.002	0.06	54	0.004	0.002	0.033	52	0.005	0.002	0.01
Whole Grains	47	−0.017	0.030	0.57	49	−0.028	0.019	0.16	47	−0.029	0.016	0.08
Fish	47	−0.014	0.010	0.15	44	−0.015	0.017	0.39	46	*	*	*
Red meat	54	0.001	0.003	0.66	51	0.003	0.004	0.53	52	0.000	0.003	0.92
Poultry and Eggs	51	0.005	0.008	0.52	54	−0.003	0.004	0.41	50	0.001	0.006	0.84
Milk and Dairy Products	54	−0.003	0.002	0.07	55	0.001	0.001	0.34	52	−0.001	0.001	0.31
Beans	52	0.005	0.003	0.15	55	−0.004	0.003	0.17	53	0.000	0.003	0.93
Vegetable Oils	54	−0.006	0.019	0.75	51	0.000	0.026	0.99	51	−0.010	0.021	0.65
Simple Sugars	53	0.022	0.008	0.006	54	0.020	0.007	0.008	52	0.009	0.007	0.19

T0—time point after the first CT cycle, T1—time point after the intermediate CT cycle, T2—time point after the last CT cycle, B—angular coefficient of linear regression equation, SE—standard error. * Variable did not comply with the prerequisite for the linear regression (the removal of outliers and extreme values made the test not viable). DII^®^ was analyzed as a continuous variable.
